# Posterior rectus sheath reattachment after eTEP rives: are we solving the right problem?

**DOI:** 10.3389/jaws.2026.17986

**Published:** 2026-10-13

**Authors:** Jorge Daes

**Affiliations:** Division of Minimally Invasive and Bariatric Surgery, Department of Surgery, Clínica Iberoamérica, Barranquilla, Colombia

**Keywords:** abdominal wall morphology, abdominal wall reconstruction, biomechanics, bulging, eTEP

## Introduction

The debate over posterior rectus sheath (PRS) reattachment after eTEP Rives repair seems straightforward: after dividing the posterior sheath, should we routinely suture it back together?

Yet the literature invoked to resolve this conundrum examines related but non-equivalent endpoints: geometry and morphology [1–5], bulging [1, 2, 4, 5], dynamic biomechanics [6], and clinical outcomes [7]. Morphology, bulging, and function are related but not equivalent endpoints. Recognizing these distinctions may be key to this debate.

## Bulging is not widening

Bulging is not a recurrence or a simple widening of the abdominal wall. It is an anterior displacement of the abdominal contour without a fascial defect [1, 2].

Clinical perception of bulging varies among patients and surgeons, making objective measurement important. Ellipse 9 was developed for that purpose and is based on two ellipses: X1 represents the overall abdominal diameter, and X2 the distance between the semilunar lines. Y2 measures anterior projection, while the c/a ratio measures eccentricity—closer to 1 is flatter; lower values indicate a rounder contour. Thus, X2 reflects transverse remodeling, whereas Y2 and c/a better characterize bulging [1, 2].

This distinction matters because X2 is not bulging. In our series, substantial X2 expansion could coexist with a flatter abdomen, whereas X2 reduction could coexist with minimal anterior rounding [1, 2].

Connors, Belyansky, and colleagues elegantly demonstrated that PRS reloading changes postoperative morphology and reduces semilunar-line separation compared with bridging [3]. This is an important anatomical observation, but bulging was not measured. They demonstrated a morphological effect; they did not demonstrate that it prevents bulging.

Halpern et al. provide complementary evidence: PRS closure produced a substantial additional reduction in semilunar-line separation, yet bulging occurred in 18.5% with closure versus 21.8% in a historical non-closure cohort (p = 0.794) [5]. This cannot establish equivalence but further separates morphological change from bulging.

## Open or closed, bulging can occur

Bulging first drew our attention after eTEP Rives for small ventral defects with diastasis; after reconstruction of larger defects, improved contour is generally expected. In series without PRS reattachment, most abdomens became flatter, although objective bulging occurred in 5 of 30 patients [1].

Rossi, Signorini, and colleagues subsequently reported a complementary finding. Despite systematic PRS reattachment, objective bulging occurred in 9 of 33 patients. Patients with bulging showed greater anterior projection and reductions in both X1- and X2-based eccentricity, producing a rounder contour [4].

Halpern et al. provide a more direct, although historical, comparison: bulging occurred in 18.5% of patients with PRS closure and 21.8% without closure (p = 0.794) [5]. This study cannot establish equivalence or determine whether closure reduces bulging.

Taken together, these studies do not establish whether PRS closure reduces the incidence of bulging.

They establish something simpler:

PRS open → bulging can occur.
PRS closed → bulging can occur.

Thus, reattachment does not prevent all bulging.

## Closure is not free

The question is not only whether PRS reattachment changes anatomy, but also what routine reattachment entails.

Approximating two divided posterior sheaths during a wide eTEP dissection can be technically demanding and time-consuming, particularly when the tissues do not meet without tension. This matters if the maneuver is to be generalized beyond surgeons and centers dedicated to advanced abdominal wall reconstruction.

Closure under tension may also fail. Posterior-layer dehiscence and subsequent intraparietal herniation, sometimes with bowel obstruction, are recognized complications of retromuscular reconstruction [8]. These risks should not be exaggerated—the recent systematic review found no significant difference in postoperative interparietal hernias between closure and non-closure—but neither should they be ignored [7].

Furthermore, reapproximating the divided PRS restores continuity of the posterior layer but does not recreate its native relationship with the linea alba, creating another postoperative geometry. Routine PRS-to-PRS reapproximation was also not a requirement of the original Rives principle [9].

The recent systematic review of 1,112 eTEP repairs is therefore particularly relevant: postoperative outcomes were broadly comparable with and without PRS closure, and the available heterogeneous evidence did not justify a standardized recommendation for closure [7].

PRS reattachment also has a prosthetic consequence. Halpern et al. demonstrated substantial narrowing of the semilunar-line separation, permitting a narrower mesh underlay [5]. When broad retromuscular mesh coverage is a reconstructive objective, this is a trade-off rather than an inherent advantage.

## A hypothesis: adaptive and maladaptive remodeling

An eTEP Rives repair does more than close a defect and place a mesh. It creates a new abdominal wall geometry. The linea alba is reconstructed, the rectus muscles change position and shape, and forces are redistributed through the reconstructed system.

Most abdominal walls appear to adapt successfully, though some may not. We hypothesize that bulging may reflect maladaptive postoperative remodeling rather than the direct consequence of leaving the PRS open.

Muscle changes may accompany—and potentially contribute to—maladaptive remodeling. We found postoperative rectus atrophy on paired CT measurements [2]. Rossi et al. independently demonstrated substantial postoperative reductions in rectus muscle density despite systematic PRS closure [4]. Dynamic MRI studies further show that abdominal wall reconstruction alters muscle geometry and dynamic displacement, with considerable interindividual variability [6].

The rehabilitation signal is intriguing but exploratory. In the final 15 patients of our series, early electrostimulation followed by progressive core training was associated with no non-stressed bulging versus 16.7% in the preceding cohort (p = 0.067), and eccentricity improved significantly (p = 0.035) [2]. The cohort was small, sequential, and nonrandomized; causality cannot be claimed.

## Beyond open versus closed

None of this means the PRS should never be reattached. If it comes together easily and safely, reapproximation is reasonable. Nor should we discount the excellent results achieved by surgeons who routinely reload the PRS.

But excellent surgical outcomes reflect the whole operation—the surgeon, technique, tissue handling, patient selection, preparation, and recovery—not necessarily a single maneuver.

We should therefore be cautious about making routine PRS reattachment the solution to a problem not shown to stem from non-reattachment.

The more interesting scientific question now lies beyond open versus closed. We need to understand how reconstruction changes geometry, muscle, and function; why most abdominal walls adapt while some do not; and whether rehabilitation can favorably influence that adaptation (Figure 1).

**FIGURE 1:**
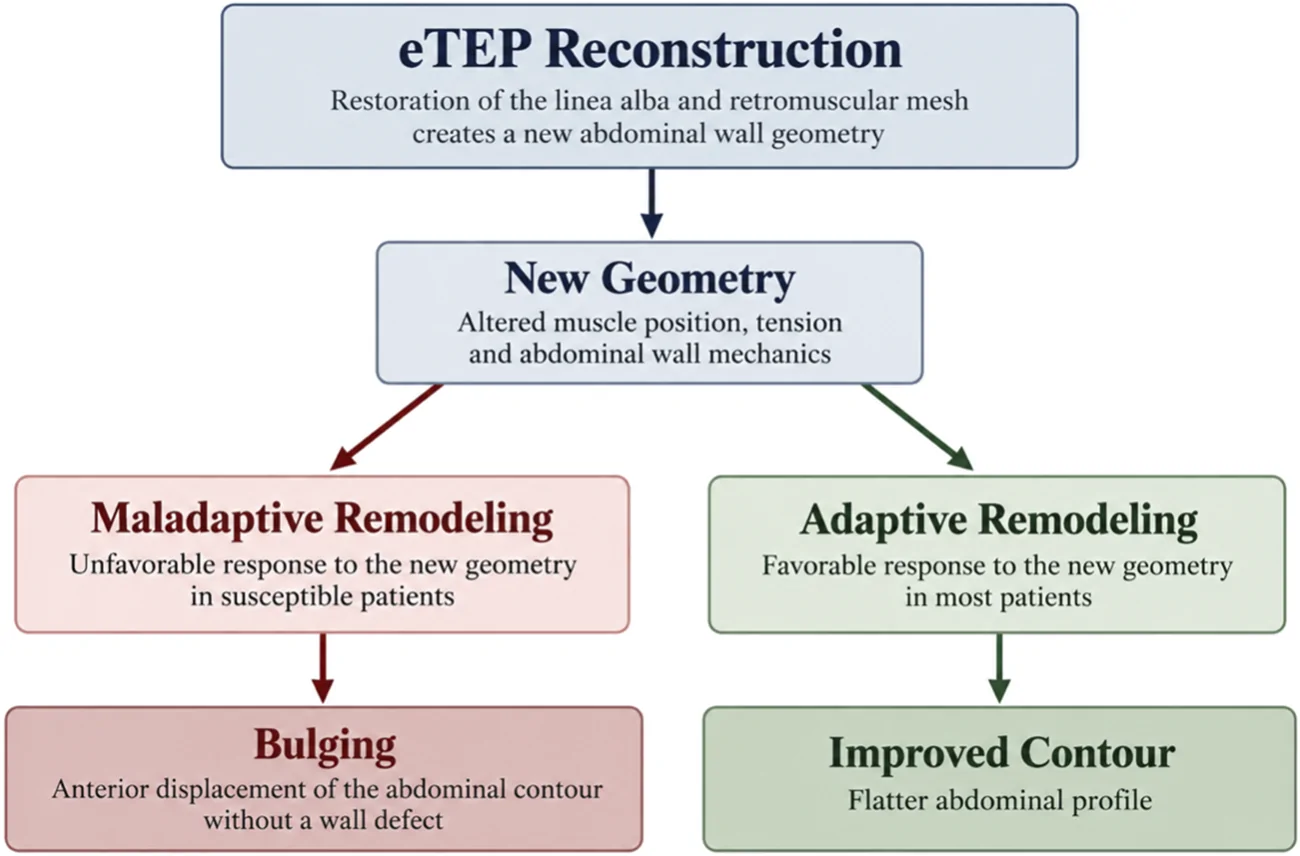
Hypothesized model of postoperative abdominal wall remodeling after eTEP Rives repair. Reconstruction creates a new abdominal wall geometry by altering muscle position, tension, and abdominal wall mechanics. Subsequent adaptive remodeling may improve contour, whereas maladaptive remodeling may lead to bulging. Multiple anatomical, muscular, and biomechanical factors likely influence these divergent responses, some of which may be modifiable through rehabilitation.

Restoring anatomy is one step. Understanding—and perhaps guiding—how the reconstructed abdominal wall adapts is the science still ahead of us.
